# Fluoride impairs vascular smooth muscle A7R5 cell lines via disrupting amino acids metabolism

**DOI:** 10.1186/s12967-024-05350-0

**Published:** 2024-06-01

**Authors:** Yan-Shu Li, Ru-Ru Yang, Xin-Ying Li, Wei-Wei Liu, Yi-Ming Zhao, Ming-Man Zu, Yi-Hong Gao, Min-Qi Huo, Yu-Ting Jiang, Bing-Yun Li

**Affiliations:** 1 School of Public Health, Shantou University, 243 Daxue Road, Jinping District, Shantou, 515063 Guangdong Province China; 2 Center for Endemic Disease Control, Chinese Center for Disease Control and Prevention, Key Lab of Etiology and Epidemiology, Harbin Medical University, Education Bureau of Heilongjiang Province & Ministry of Health (23618504), Harbin, 150081 China; 3 Weihai Municipal Hospital, Weihai, 264299 Shandong Province China; 4 Xinyi Center for Disease Control and Prevention, Xinyi, China

**Keywords:** Cardiovascular, Metabolomics, Amino acids metabolism

## Abstract

Given the insidious and high-fatality nature of cardiovascular diseases (CVDs), the emergence of fluoride as a newly identified risk factor demands serious consideration alongside traditional risk factors. While vascular smooth muscle cells (VSMCs) play a pivotal role in the progression of CVDs, the toxicological impact of fluoride on VSMCs remains largely uncharted. In this study, we constructed fluorosis model in SD rats and A7R5 aortic smooth muscle cell lines to confirm fluoride impaired VSMCs. Fluoride aggravated the pathological damage of rat aorta in vivo. Then A7R5 were exposed to fluoride with concentration ranging from 0 to 1200 μmol/L over a 24-h period, revealing a dose-dependent inhibition of cell proliferation and migration. The further metabolomic analysis showed alterations in metabolite profiles induced by fluoride exposure, notably decreasing organic acids and lipid molecules level. Additionally, gene network analysis underscored the frequency of fluoride's interference with amino acids metabolism, potentially impacting the tricarboxylic acid (TCA) cycle. Our results also highlighted the ATP-binding cassette (ABC) transporters pathway as a central element in VSMC impairment. Moreover, we observed a dose-dependent increase in osteopontin (OPN) and α-smooth muscle actin (α-SMA) mRNA level and a dose-dependent decrease in ABC subfamily C member 1 (ABCC1) and bestrophin 1 (BEST1) mRNA level. These findings advance our understanding of fluoride as a CVD risk factor and its influence on VSMCs and metabolic pathways, warranting further investigation into this emerging risk factor.

## Background

Fluoride is naturally abundant in the earth's crust and continuously forms in soil and aquifer sediments [24]. As a result, humans often unknowingly consume excessive amounts of fluoride through water and various food sources, and fluorosis is more prevalent in areas where water fluoridation cannot be consistently monitored. According to the World Health Organization guideline, which recommends a fluoride concentration of 1.5 mg/L in drinking water, an estimated 63–330 million people are at risk of exposure to fluoride levels exceeding this threshold [32]. Furthermore, in the Eastern Africa Rift Valley System, the mean fluoride content in foods like potatoes, beans, and peas has surpassed the recommended dietary allowances level of 4 mg/kg endorsed by the US Institute of Medicine. This elevated fluoride intake may pose a health threat, particularly to young children, male populations, or individuals engaged in high levels of physical activity [26]. Notably, fluoride primarily accumulates in the body and predominantly travels through the bloodstream, with blood fluoride concentrations peaking within one hour after ingestion. Importantly, this process is not constrained by the total fluoride mass in the body [5, 16]. In addition to causing dental and skeletal fluorosis, high levels of fluoride exposure from drinking water (F^−^ ≥ 3.01 mg/L) has been linked to cardiovascular diseases (CVDs). The residents living in fluoride endemic areas for at least 10 years had higher risk of suffering impaired aortic elasticity, hypertension, and carotid atherosclerosis [37, 38, 44]. Given the stealthy nature and high fatality rate of CVDs, it is imperative to take the newly identified risk of fluoride seriously, alongside conventional risk factors.

When vascular injury occurs or the vascular microenvironment undergoes changes, even subtle shear stress can trigger a transition in vascular smooth muscle cells (VSMCs). These cells shift from a quiescent contractile phenotype to a highly mobile and proliferative synthetic phenotype, a process that forms the pathological foundation of vascular diseases [7, 10, 35]. Emerging evidence suggests a strong correlation between VSMC metabolism and phenotype switching, as well as CVDs such as hypertension and atherosclerosis [9, 45]. Glucose metabolism is pivotal for all organisms with regard to VSMC function. In rat aortic smooth muscle cell lines (A7R5), the overexpression of glucose transporter 1 (GLUT1) leads to an increased flux of glucose through glycolysis and the tricarboxylic acid (TCA) cycle, promoting VSMCs proliferation via producing ATP [20, 53]. Glycolysis, as the primary metabolic pathway, accounts for over 90% of glucose utilization in VSMCs. Key glycolytic enzyme hexokinase 2 (HK2) plays a crucial role in regulating glycolytic activity and the transition of VSMCs phenotypes in vitro [43, 47, 49]. Additionally, the composition of fatty acids, essential components of cell structure and function, can impact VSMCs in terms of proliferation, migration, and differentiation. The elongation of long-chain fatty acid member 6 (Elovl6), which increases palmitate and reduces oleate, regulates VSMC phenotype switching through reactive oxygen species (ROS) and AMP-activated protein kinase (AMPK) [39]. Palmitic acid also inhibits the transition of VSMCs to a synthetic phenotype by upregulating miR-22, highlighting the potential of correcting fatty acid metabolism as a treatment approach for vascular diseases [13]. Furthermore, studies have shown that amino acids, such as glutamine and arginine, participate in maintaining cellular redox balance in VSMCs [28, 30]. In cases of metabolic syndrome, certain molecules like fatty acids, leptin, and oxidized lipoprotein particles induce VSMC proliferation by activating the generation of ROS [23]. These observations suggest that minor alterations in metabolites are intricately linked to VSMC functionality.

Metabolomics serves as a rapid and highly sensitive approach for the investigation of a multitude of differentially abundant metabolites (DAMs). While the toxic effects of fluoride on serum [19, 50], tissues [52] and cells [51] are gradually emerging, there is a notable absence of information regarding the metabolites in VSMCs exposed to fluoride. This study seeks to uncover changes in metabolites within VSMCs treated with fluoride, with the hope of offering novel insights and potential avenues for future research, possibly leading to more effective therapies.

## Methods

### Animals, treatments and fluoride determination

40 male 3-week-old Sprague–Dawley rats were purchased from Beijing Vital River Laboratories (Beijing, China) and were allowed to acclimatize for a week in a humidity-controlled room with a 12 h light/dark cycle. The rats have free access to the maintenance fodder (Beijing Vital River Laboratories) and sterilized distilled water provided by animal feeding institution. Subsequently, these rats were randomly divided into 4 groups, which were control group (sterilized distilled water), 50 mg/L, 100 mg/L, 150 mg/L fluorinated water, respectively. After 24 weeks of feeding, thoracic aortas were obtained and fixed with 10% methanal for pathological examination. Urine of rats was measured by fluoride ion selective electrode method [22] to reflect fluoride exposure in rats. All animal procedures were approved by the Animal Care and Use Committee of Harbin Medical University, Harbin, China (NO. hrbmuecdc20230501).

### Masson staining

The fixed vascular tissue was dehydrated and transparent in gradient alcohol and xylene. Afterwards, they were embedded in paraffin and sectioned (6 μm) by a microtome. Masson staining kit (Solarbio life sciences, Beijing, China) was used to observe the damage of vascular smooth muscle layer. Olympus microscope was used to capture the images of vascular masson staining slices.

### Cells viability

Rat aortic smooth muscle cell lines (A7R5) were procured from Meisen Chinese Tissue Culture Collections (Meisen CTCC) and maintained in Dulbecco's modified Eagle's medium (DMEM) supplemented with 10% fetal bovine serum (FBS, GIBCO) and 1% antibiotic–antimycotic (GIBCO), with incubation at 37 °C in a 5% CO_2_ atmosphere.

During the logarithmic growth phase, VSMCs were seeded at a density of 5000 cells per well in 96-well plates and allowed to adhere for 24 h. Following this, the VSMCs were subjected to various concentrations of NaF for 24 h. The original culture medium was subsequently removed, and each well received 200 µL of 10% CCK-8 reagent. The plate was then incubated at 37 °C for 30 min, and absorbance was measured at 450 nm (Bio Tek Instruments Inc., Highland Park, Vermont, USA). Cell viability was expressed as a percentage relative to untreated control cells, calculated using the following formula: Cell viability (%) = (OD450 of experimental groups—OD450 of the blank group) / (OD450 of the control group—OD450 of the blank group) × 100%.

### Scratch wound healing assay

Cells were initially seeded at a density of 5 × 10^5^ cells per well in a 6-well culture plate and cultured for an additional 24 h until they reached approximately 80% confluence. Subsequently, a straight-line scratch was made across the plate using a sterilized 10-μL pipette tip. After thorough washing with DMEM, the cells were incubated for 24 h at 37 °C in DMEM containing various concentrations of NaF and 2% FBS. Images of the scratched cells were captured at both 0 h and 24 h using a microscope (U-LH100-3, OLYMPUS). The wound area was quantified using ImageJ (NIH, USA), and the extent of migration was determined by measuring the gap closure area between the 0-h and 24-h time points. The calculation formula for assessing cell migration ability was as follows: closed wound area (%) = (0-h area—24-h area) / 0-h area × 100%.

### Metabolomic analysis

Until A7R5 reached approximately 80% confluence, the cells which were treated with DMEM and fluoridated DMEM for 24 h were first rinsed twice with pre-cooled PBS (n = 6). Subsequently, the cells were detached by adding an appropriate amount of trypsin and incubating for 2 min to collect them into a centrifuge tube. After centrifugation at 1000 g and 4 °C for 1 min, the cell pellet was promptly quenched with liquid nitrogen. Quality control (QC) samples were prepared by pooling 10 µL of each sample and analyzed together with the other samples to correct any deviations in the test system.

For the LC–MS/MS analysis, we utilized a UHPLC system (Agilent Technologies 1290 Infinity LC) coupled to a quadrupole time-of-flight mass spectrometer (AB Sciex TripleTOF 6600) at Shanghai Applied Protein Technology Co., Ltd. HILIC separation was performed using a 2.1 mm × 100 mm ACQUITY UPLC BEH Amide column with 1.7 µm particle size (Waters, Ireland). In both ESI positive and negative ionization modes, the mobile phase consisted of A (25 mM ammonium acetate and 25 mM ammonium hydroxide in water) and B (acetonitrile). The gradient started at 95% B for 0.5 min, then gradually decreased to 65% over 6.5 min, followed by a reduction to 40% in 1 min, maintained for 1 min, and finally raised back to 95% in 0.1 min, with a 3-min re-equilibration period.

The ESI source conditions were configured as follows: Ion Source Gas1 (Gas1) at 60, Ion Source Gas2 (Gas2) at 60, curtain gas (CUR) at 30, source temperature at 600 °C, and IonSpray Voltage Floating (ISVF) at ± 5500 V. In MS-only acquisition, the instrument was set to scan the m/z range from 60 to 1000 Da, with an accumulation time of 0.20 s per spectrum. For the auto MS/MS acquisition, the instrument covered the m/z range from 25 to 1000 Da, with an accumulation time of 0.05 s per spectrum for the product ion scan. The product ion scan was performed using information-dependent acquisition (IDA) in high sensitivity mode. The parameters for IDA were as follows: collision energy (CE) was set at a fixed value of 35 V with ± 15 eV; declustering potential (DP) at 60 V for positive mode and − 60 V for negative mode; exclusion of isotopes within 4 Da, and 10 candidate ions monitored per cycle.

### Quantitative real-time PCR

Until A7R5 reached approximately 80% confluence, the cells which were treated with DMEM and fluoridated DMEM for 24 h were used to extract total mRNA by TRIZOL reagent. Quantitative real-time PCR (qRT-PCR) analysis was carried out to evaluate RNA expression in control and fluoride treated A7R5 (n = 3). cDNA was synthesized with the Prime Script RT Reagent Kit with gDNA Eraser reverse transcriptase (Takara Bio, Dalian, China). qRT-PCR reactions were conducted with the SYBR Green kit (Takara). Data were normalized to GAPDH as the reference genes for mRNAs. The primer sequences used are provided as followed: ACTCCCATTCTTCCACCTTTG and CCCTGT.

TGCTGTAGCCATATT for GAPDH mRNA; AGGAGTTTCCCTGTTTCTGATG and GCAACTGGGATGACCTTGATA for OPN mRNA; AAGGACAGCTATGTGGGGGA and CGTTAGCAAGGTCGGATGCT for α-SMA mRNA; TGCAAGCAGGCTTTATGACTC and CAGTGTCCCTGATTCGACCT for BEST1 mRNA; ATTGTCATGAGTGGCGGCAA and CTTCCCTAAACCACTGACACCA for ABCC1 mRNA.

### Statistics

The raw metabolomics data were initially processed using ProteoWizard MSConvert, followed by subsequent analysis utilizing the R package (ropls). The processed data underwent multivariate data analysis, encompassing Pareto-scaled principal component analysis (PCA) and orthogonal partial least-squares discriminant analysis (OPLS-DA). The Variable Importance in the Projection (VIP) value was computed for each variable within the OPLS-DA model to assess its contribution to the classification. Metabolites with VIP value exceeding 1 and P-value less than 0.05 were identified as significantly altered. Quantitative data were presented as the mean ± standard deviation (SD). To determine the significance of differences among multiple groups of independent samples, one-way Anova was employed. The significance level was set at *P* < 0.05 to establish statistical significance and statistics were calculated with SPSS 19.0.

## Result

### Fluoride exposure induces pathological alteration of rat aortic media

**Fig. 1:**
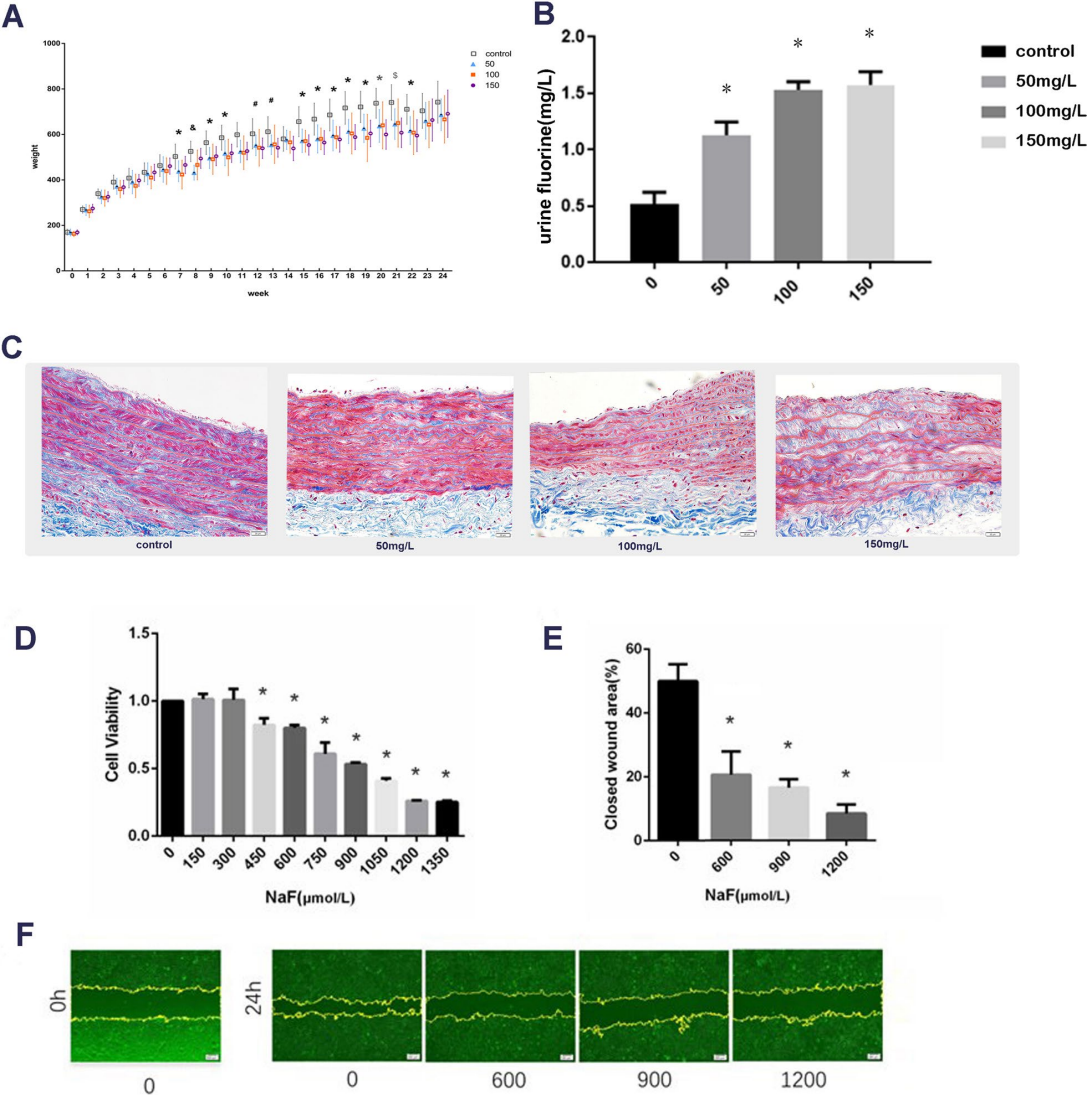
Fluoride impacted on VSMCs in vivo and in vitro. **A** Rats weight during feeding period. Compared with the control group, * represents all three fluoride treated groups, # represents 150 mg/L fluoride treated group, $ represents 50 and 150 mg/L fluoride treated groups, and & represents 50 and 100 mg/L fluoride treated groups. **B** Fluoride exposure in rats. * *P* < 0.05 versus control. **C** Pathological alteration of rats’ aortas media with masson staining. **D** Cell viability assay. VSMCs were treated with various concentrations of NaF for 24 h. Cell viability was assessed by CCK-8 cell proliferation kit. (*P < 0.05 versus control, n = 4). **E** Scratch wound assay. Data were quantified by measuring the close area in (**F**) (*P < 0.05 versus control, n = 3), and presented as mean ± SD. **F** Representative images of VSMCs scratch wound assay. The closed wound area was quantified by ImageJ (scale bar, 200 µm)

As shown in Fig. 1A, the body weight of rats in the fluoride treatment groups was lower than that in the control group after 7 weeks feeding, which showed that fluoride had a certain toxic effect on the growth of rats. The fluorine concentration in 150 mg/L, 100 mg/L and 50 mg/L groups were significantly higher than that in the control group (*P* < 0.05), which indicating that fluorine must be higher than urine fluorine concentration when flowing through blood vessels.

In the control group, VSMCs in the media of rat aorta were well organized and elastic fiber were integrated and continuous. It could be seen from data that with fluoride exposure increasing, the media layer showed progressive disordered VSMCs arrangement, decreased elastic fiber integrity and suspected increased vascular wall fragility (Fig. 1C). In short, fluoride aggravated the pathological damage of rat thoracic aorta.

### Fluoride inhibits VSMC proliferation and wound healing

To identify the optimal fluoride concentration for establishing the cell model, VSMCs were exposed to 0–1350 μmol/L NaF concentrations for 24 h. After treatment with 600, 900 and 1200 μmol/L fluoride for 24 h, the proliferation of VSMC was inhibited to 80%, 53% and 26%, respectively (Fig. 1D). Vascular smooth muscle cell apoptosis is the driving factor of vascular diseases, in view of apoptosis and survival, NaF dose at 900 μmol/L was implemented in metabolomic analysis and 600, 900 and 1200 μmol/L fluoride was used in wound healing and qRT-PCR.

With the increase of fluoride concentration, the wound healing area gradually decreased, and the scratch healing rates of 0, 600, 900 and 1200 μmol/L fluoride treatment groups were 50%, 21%, 17% and 8%, respectively, indicating that fluoride had an inhibitory effect on the migration ability of VSMC (Fig. 1E, F).

### Metabolomics data quality

**Fig. 2:**
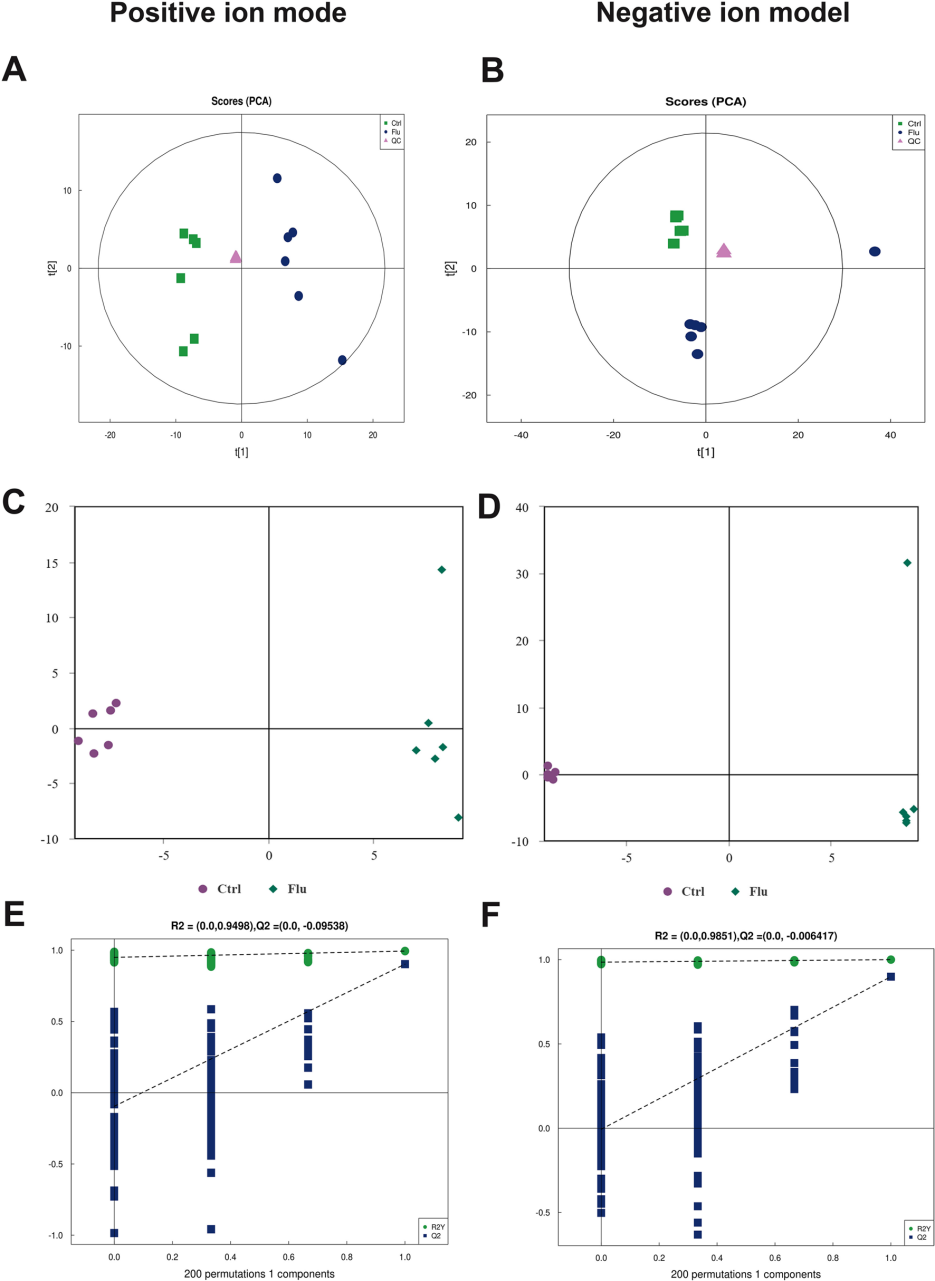
Multidimensional data analysis. PCA score plots (**A**, **B**) of cellular metabolic profiles in positive and negative ion mode. OPLS-DA score plots (**C**, **D**) and curves (**E**, **F**) in positive and negative ion mode

Both the experimental samples and the quality control (QC) samples underwent PCA, as depicted in Fig. 2A, B. The R2X values in positive ion model (PIM) and negative ion model (NIM) were calculated to be 0.558 and 0.607, respectively. These values demonstrated that the QC samples formed tight clusters, indicating the experiment's excellent repeatability. Furthermore, OPLS-DA plots (Fig. 2C, D) and curves (Fig. 2E, F) exhibited R2Y and Q2 values of 1 and 0.899 in NIM, and 0.994 and 0.902 in PIM. These values signify the robust predictive capability of our model and indicate that it was not overfitted.

### Identification of fluoride treated VSMC metabolites

**Fig. 3:**
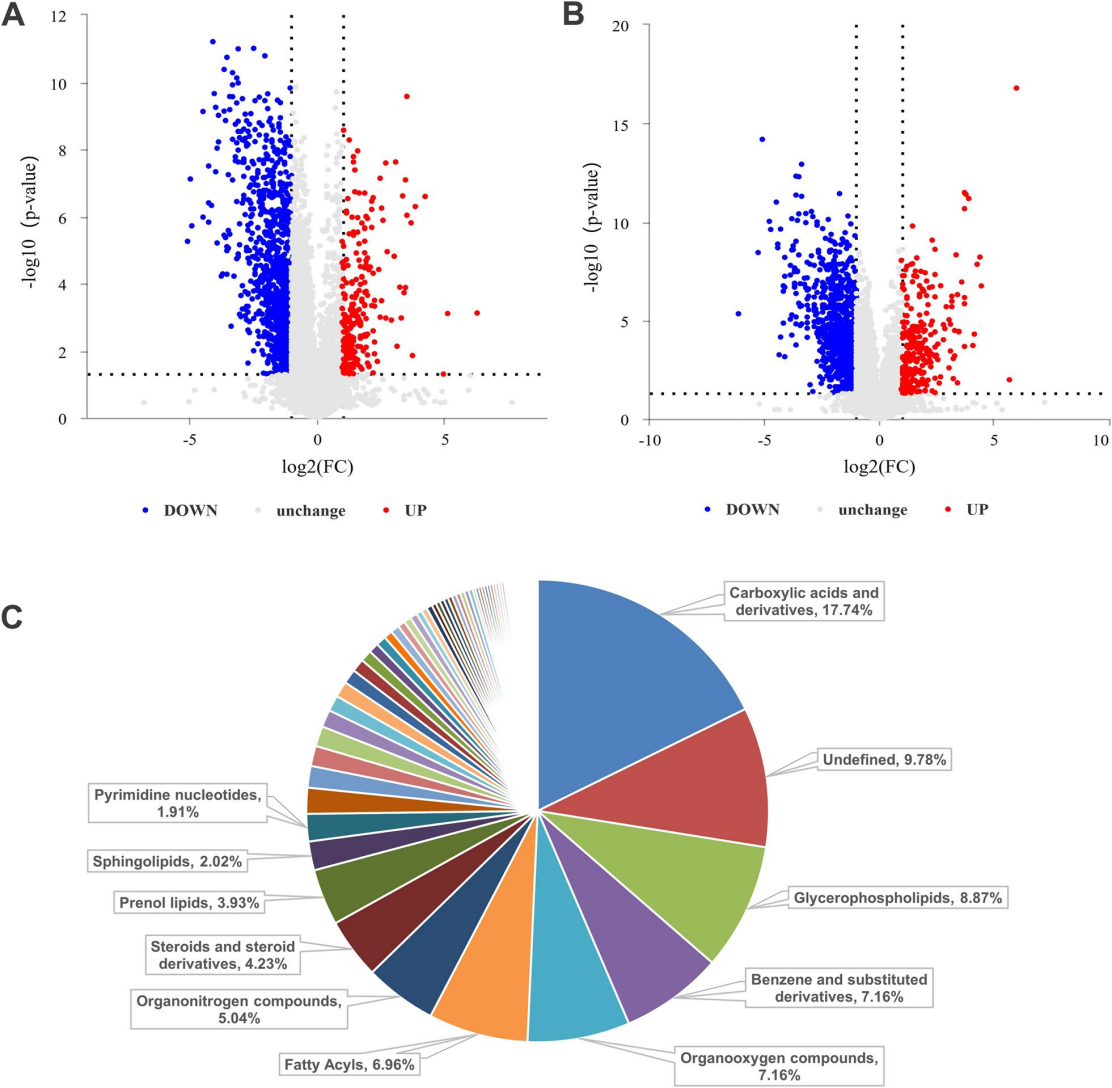
Metabolites identification. **A** and **B** Deregulation of metabolites detected in positive ion mode and negative ion mode. **C** Classification of different identified metabolites. The percentage represents the number of metabolites attribution entry as a percentage of all metabolites

A comparison between the control group and the fluoride-treated group revealed a large majority of metabolites were identified in PIM and NIM as shown in Fig. 3A, B. The screening criteria for significance were defined as fold change log2 (FC) > 2 and P < 0.05. Next, the metabolites of VSMC treated by fluoride were analyzed, and the top 10 categories of DAMs were successfully identified, with carboxylic acids and derivatives constituting the largest category at 17.742%, followed by glycerophospholipids at 8.87%, benzene and substituted derivatives at 7.157%, organooxygen compounds at 7.157%, fatty acyls at 6.956%, organonitrogen compounds at 5.04%, steroids and steroid derivatives at 4.234%, prenol lipids at 3.931%, and sphingolipids at 2.016% (Fig. 3C).

### Fluoride mainly disturbed VSMCs amino acids metabolism

**Fig. 4:**
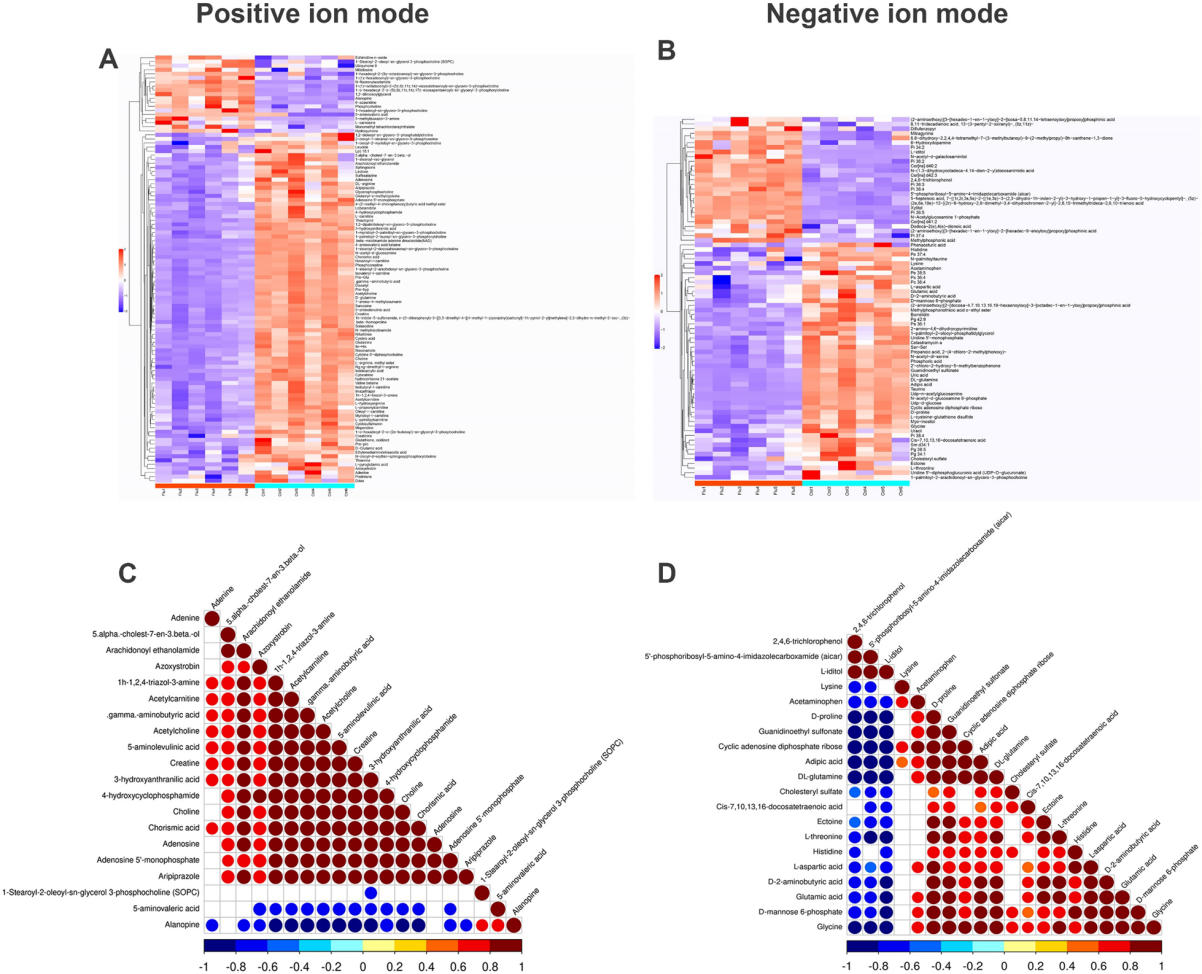
Hierarchical clustering and Correlation analysis. **A** and **B** Heatmap of the hierarchical clustering of differentially abundant metabolites between fluoride treat group and the control group. Flu, fluoride group; Ctr, control group. N = 6. **C** and **D** are top 20 differential metabolites correlation in fluoride exposed group in positive ionization mode and negative ionization mode, respectively. Red represents positive correlation and blue represents negative correlation

To observe the hierarchical clustering results visually, we conducted a heat map to represent, where each column corresponds to a sample and each row represents a specific metabolite. The majority of metabolites in both the fluoride-treated and control groups exhibit well-defined clustering patterns among the samples (Fig. 4A, B). Among all identified metabolites, 16 metabolites displayed upregulation and 80 metabolites exhibited downregulation, excluding undefined ones in PIM. In NIM, 21 were found to be upregulated, while 46 were downregulated.

Following the exclusion of undefined metabolites, a correlation analysis was conducted to examine the relationships among the top 20 metabolites. In PIM, specifically among organic oxygen compounds (amino acids), 5-aminovaleric acid and alanopine exhibited negative correlations with other metabolites such as amino acids, lipids, nucleosides, and organic nitrogen compounds (Fig. 4C). Conversely, numerous amino acids including glutamic acid, glycine, D-2-aminobutyric acid, L-aspartic acid, histidine, L-threonine, ectoine, D-proline, DL-glutamine, D-proline, and lysine displayed negative correlations with 5’-phosphoribosyl-5-amino-4-imidazolecarboxamide (AICAR) in NIM. Additionally, there were noticeable positive correlations among these amino acids (Fig. 4D). It was also observed that, irrespective of the ionization mode, a significant number of DAMs were associated with major energy metabolism processes in fluoride-exposed VSMCs, as detailed in Tables 1 and 2. Notably, amino acids took the largest proportion among all energy metabolism related metabolites.

**Table 1:** Fluoride exposed disturbed VSMCs energy metabolism in positive ion mode

SubClass	Name	HMDB	VIP	p-value
Amino acids, peptides, and analogues	Creatine	HMDB0000064	20.800	< 0.001
	5-aminovaleric acid	HMDB0003355	9.416	0.008
	L-hydroxyarginine	HMDB0004224	8.492	< 0.001
	D-glutamine	HMDB0003423	8.374	< 0.001
	DL-arginine		6.802	< 0.001
	Pro-hyp	HMDB0006695	6.104	< 0.001
	L-pyroglutamic acid	HMDB0000267	5.625	0.011
	Sarcosine	HMDB0000271	3.915	< 0.001
	.gamma.-aminobutyric acid	HMDB0000112	3.473	< 0.001
	Leucine	HMDB0000687	3.030	0.002
	Glutamine	HMDB0000641	3.005	< 0.001
	Phosphocreatine	HMDB0001511	2.848	< 0.001
	Creatinine	HMDB0000562	2.584	0.001
	Cysteic acid	HMDB0002757	2.530	< 0.001
	Imazethapyr		1.968	< 0.001
	Ng,ng-dimethyl-l-arginine	HMDB0001539	1.744	< 0.001
	Valine betaine		1.623	< 0.001
	5-aminolevulinic acid	HMDB0001149	1.479	< 0.001
	D-Glutamic acid	HMDB0003339	1.376	< 0.001
	Alanopine	HMDB0033747	1.369	< 0.001
	Pro-pro		1.334	0.001
	Glutathione, oxidized	HMDB0003337	1.244	0.002
	Glutamyl-s-methylcysteine	HMDB0031985	1.240	0.001
	Pro-Glu		1.159	< 0.001
	Ile-His		1.116	< 0.001
	L-arginine, methyl ester		1.093	< 0.001
Carbohydrates and carbohydrate conjugates	Lactose	HMDB0000186	5.384	0.001
	N-acetyl-d-glucosamine	HMDB0000215	2.869	< 0.001
	6-azauridine		2.678	< 0.001
Fatty acid esters	Acetylcarnitine		8.347	< 0.001
	L-palmitoylcarnitine	HMDB0000222	6.133	< 0.001
	Oleoyl-l-carnitine	HMDB0005065	5.165	< 0.001
	L-propionylcarnitine	HMDB0062514	4.227	< 0.001
	Myristoyl-l-carnitine	HMDB0005066	2.736	< 0.001
	Isovaleryl-l-carnitine		2.650	< 0.001
	Isobutyryl-l-carnitine	HMDB0000736	2.408	< 0.001
	Hexanoyl-l-carnitine	HMDB0000756	1.483	< 0.001
Fatty acids and conjugates	4-aminovaleric acid betaine		4.560	< 0.001

*VIP* variable important in projection

P < 0.05 versus the control group (n = 6)

**Table 2:** Fluoride exposed disturbed VSMCs energy metabolism in negative ion mode

SubClass	Name	HMDB	VIP	p-value
Amino acids, peptides, and analogues	DL-glutamine		5.781	< 0.001
	Glutamic acid	HMDB0000148	4.836	0.004
	Ectoine		3.422	0.047
	D-proline	HMDB0003411	2.668	< 0.001
	L-threonine	HMDB0004041	2.240	0.004
	L-aspartic acid	HMDB0062186	1.980	0.013
	D-2-aminobutyric acid	HMDB0000452	1.542	0.005
	Lysine	HMDB0003405	1.534	0.005
	Histidine	HMDB0000177	1.233	0.033
	L-cysteine-glutathione disulfide		1.209	< 0.001
	Glycine	HMDB0000123	1.170	< 0.001
	Phenaceturic acid	HMDB0000821	1.074	0.041
	Ser-Ser		1.071	< 0.001
Carbohydrates and carbohydrate conjugates	Xylitol	HMDB0002917	3.841	< 0.001
	L-iditol	HMDB0011632	3.705	< 0.001
	D-mannose 6-phosphate	HMDB0001078	2.256	0.002
	Cyclic adenosine diphosphate ribose		1.903	< 0.001
	N-Acetylglucosamine 1-phosphate	HMDB0001367	1.783	< 0.001
	N-acetyl-d-galactosaminitol		1.343	< 0.001
	N-acetyl-d-glucosamine 6-phosphate	HMDB0001062	1.332	< 0.001
Fatty acids and conjugates	Cis-7,10,13,16-docosatetraenoic acid	HMDB0002226	4.202	0.018
	5-heptenoic acid, 7-[(1r,2r,3 s,5 s)-2-[(1e,3 s)-3-(2,3-dihydro-1 h-inden-2-yl)-3-hydroxy-1-propen-1-yl]-3-fluoro-5-hydroxycyclopentyl]-, (5z)-		3.048	< 0.001
	Dodeca-2(e),4(e)-dienoic acid		1.911	0.007
	Adipic acid	HMDB0000448	1.814	< 0.001
	8,11-tridecadienoic acid, 13-(3-pentyl-2-oxiranyl)-, (8z,11z)-		1.183	0.023

VIP, variable important in projection. P < 0.05 versus the control group (n = 6)

### DAGs are enriched most in ABC transporters pathway in fluoride treated group

**Fig. 5:**
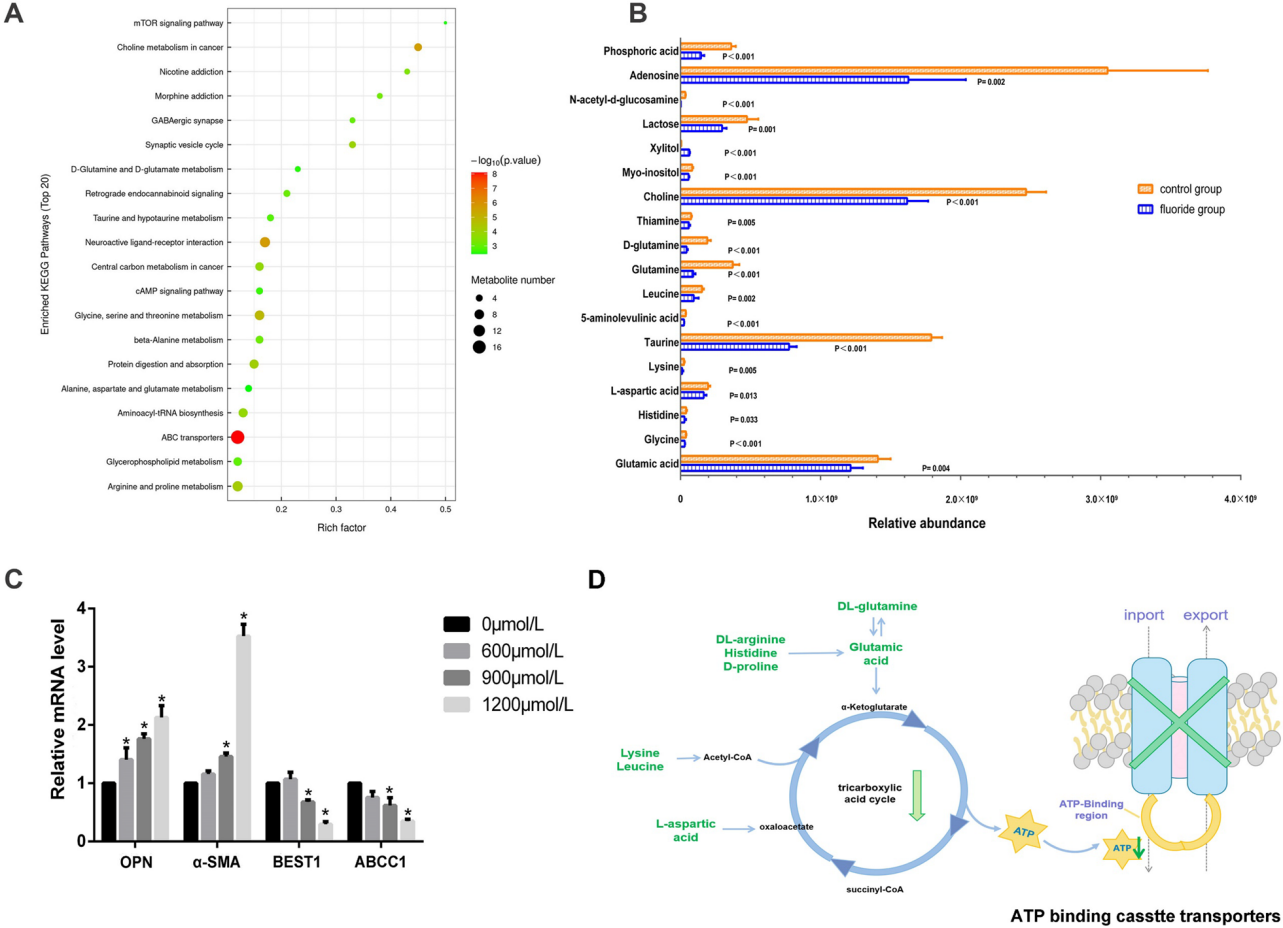
ABC transporters is the most enriched pathway in fluoride treatment group. **A** The top 20 perturbed metabolic pathways enriched by KEGG. **B** Relative abundance of DAMs enriched in ABC transporters. **C** Relative mRNAs level in fluoride-treated VSMCs. *P < 0.05, versus control group. **D** Proposed link between amino acids metabolism disturbance and ABC transporters. Eight amino acids in green fonts were downregulated in fluoride treated group. Downward green arrows indicate downregulated efficiency of TCA cycle

In order to clarify these complex metabolites, the top 20 perturbed metabolic pathways identified in the Kyoto Encyclopedia of Genes and Genomes (KEGG) are presented in Fig. 5A. Notably, amino acids are frequently enriched in many of these pathways, such as ATP-binding cassette transporters (ABC transporters), arginine and proline metabolism, glycerophospholipid metabolism, alanine, aspartate and glutamate metabolism, glycine, serine and threonine metabolism, etc., which are among those significantly impacted (Fig. 5A). Furthermore, we presented relative abundance of all DAMs enriched in ABC transporters pathway in Fig. 5B. Moreover, we detected relative mRNA level of osteopontin (OPN), α-smooth muscle actin (α-SMA), bestrophin 1 (BEST 1) and ATP binding cassette subfamily C member 1 (ABCC1) as an initial validation. Strikingly, compared to the control group, we observed a dose-dependent increase in OPN and α-SMA mRNA level and a dose-dependent decrease in BEST1 and ABCC1 mRNA level (P < 0.05). Altogether, the data suggested that ABC transporters might be involved in modulating VSMCs phenotype switch.

## Discussion

This study employed in vivo and in vitro experiments investigate the adverse effects of fluoride on VSMCs. Metabolomics analysis showed a considerable number of DAMs, classified into categories such as lipid and lipid-like compounds, organic acids and derivatives, organic oxygen compounds, and organoheterocyclic compounds, were identified in fluoride-treated VSMCs. A comprehensive analysis of these differential metabolites and enriched pathways revealed that fluoride most frequently disrupts amino acids metabolism in VSMCs. For the first time, this study demonstrates that fluoride primarily hinders VSMCs' proliferation and migration by impeding amino acids metabolism, stemming from disturbances in ABC transporters.

Amino acids metabolism, serving as the foundation for protein synthesis, plays a crucial role by providing essential structural elements and energy sources for biosynthetic reactions [21]. Previous studies have reported that fluoride exposure can lead to a reduction in amino acids in the heart, liver, and kidney of rats. Specifically, in the heart tissue of rats, L-serine, L-glutamine, L-aspartic acid, and L-glutamic acid exhibited decreased levels [52]. Consistent with these findings, among the DAMs identified in the fluoride-exposed group, a total of 39 metabolites in both PIM and NIM were found to be involved in amino acids metabolism. Notably, several of these amino acids, including glutamine, glutamic acid, proline, histidine, and arginine, are categorized within the "glutamate family" and are metabolically connected through conversion to glutamate [40]. Glutamine serves as a pivotal link between carbohydrate and protein carbon metabolism and acts as a primary nitrogen source. Furthermore, glutamine and glutamate participate in the TCA cycle in many forms and are essential constituents of the antioxidant glutathione [42]. It has shown that supplementation with glutamic acid significantly increases protein synthesis in primary cultures of hepatocytes from elderly rats, effectively doubling the rate in comparison to elderly rats not receiving glutamic acid supplementation [4]. Additionally, glutathione, a well-known antioxidant, plays a crucial role in combating free radicals to mitigate oxidative stress. In this study, it was observed that the levels of glutathione and metabolites from the "glutamate family" in fluoride-exposed VSMCs were lower than those in the control group, signifying a reduced TCA cycle activity and an elevated oxidative state.

Furthermore, arginine is essential in vascular function as it serves as the substrate for endothelial nitric oxide synthase to generate nitric oxide (NO). This process involves the transportation of arginine into VSMCs via the cationic amino acid transporter family, where it can be metabolized to form NO, polyamines, or L-proline [2]. When there is a reduction in vascular NO production or when the cyclic guanosine monophosphate-protein kinase G pathway becomes impaired, the vasodilatory function of NO in both macro- and micro-vessels is compromised [1]. This underscores the critical role of arginine and NO in regulating vascular health and function.

Notably, this study has also revealed a notable decrease in two novel amino acids in fluoride exposed VSMCs. One of these, valine betaine, is a novel betainized compound in humans, and its concentration was substantially reduced in fluoride treated VSMCs. In studies involving mice, the concentration of valine betaine has shown an inverse correlation with postprandial insulin levels [17]. It's well-established that serum insulin levels can influence VSMC phenotype alterations, proliferation, or apoptosis [6, 11, 31]. Additionally, taurine, another amino acids with anti-inflammatory properties [33], has been associated with protective effects against coronary heart disease [46] and CVDs [3]. Taurine's mechanisms of action include the maintenance of mitochondrial respiratory function and the reduction of superoxide production [34]. Therefore, fluoride impacts various amino acids in VSMCs, affecting processes like the citric acid cycle, oxidative processes, and other cellular biosynthetic pathways, which can directly or indirectly inhibit VSMC function.

Acetyl-CoA holds significant importance in the TCA cycle, and it can be generated through both glucose and fatty acid metabolism. Acetyl-CoA serves not only as the product of the oxidative decarboxylation of pyruvate but also as the carbon source for lipoic acid synthesis, cholesterol synthesis, and acetone body formation. Glycolysis plays a critical role in activating VSMCs, and its end product, pyruvate, can be converted into lactate or further metabolized to generate acetyl-CoA [12]. It's worth noting that thiamine facilitates the conversion of pyruvate to acetyl-CoA, serving as an initiator of the TCA cycle [15]. In this study, we observed decreases in thiamine, lactate, N-acetyl-d-glucosamine (NAG), and D-mannose 6-phosphate in the fluoride-treated group, suggesting an impact of fluoride on VSMC glycolysis. Due to sodium fluoride is a direct inhibitor of the glycolytic enzyme enolase, the relationship between fluoride level and VSMC glycolysis need further research. Within the enriched lipids and lipid-like molecules, acetylcarnitine was identified as decreased in the fluoride-exposed group and was associated with the insulin resistance pathway. Acetylcarnitine plays a role in transferring two-carbon units from mitochondria to the cytosol, supporting acetylation of acetyl-CoA, fatty acid synthesis, and cell growth [14]. Collectively, in fluoride exposed VSMCs, the most enriched metabolites belong to the amino acid metabolism category, which is closely interconnected with the TCA cycle. Furthermore, several DAMs related to glucose and lipid metabolism point to an impact on acetyl-CoA levels. The TCA cycle is intimately linked to ATP production and mitochondrial function, and fluoride exposure can induce morphological damage in rat cardiomyocytes (H9c2), leading to reduced concentrations of both Ca^2+^ and mitochondrial numbers. These findings underscore the intricate mechanisms through which fluoride can disrupt cellular metabolism and function.

In this study, a majority of the KEGG enrichment pathways are closely associated with amino acids metabolism. Pathways such as arginine and proline metabolism, glycine, serine, and threonine metabolism, alanine, aspartate, and glutamate metabolism, histidine metabolism, and arginine biosynthesis is intricately linked to different stages of protein synthesis. Moreover, ABC transporters, recognized as one of the largest superfamilies of conserved proteins, play a pivotal role in actively translocating various molecules, including fatty acids, amino acids, lipids, and proteins [41]. These transporters are distributed across a wide range of cellular and organelle membranes, including peroxisomes, mitochondria, lysosomes, and the endoplasmic reticulum. Peroxisomal ABC transporters, in particular, facilitate the entry of diverse lipid substrates into peroxisomes, where they are primarily subjected to degradation but also contribute to the synthesis of bioactive lipids that impact membrane composition and signaling pathways. It is worth noting that certain studies have suggested that the secretion of aminoacyl-tRNA synthetases underlies angiogenic responses, although reliable assays for assessing their specific roles in angiogenesis functions are still lacking [25]. These findings emphasize the complexity of the metabolic pathways and molecular mechanisms at play in VSMCs exposed to fluoride.

In addition, increased synthetic phenotype marker gene α-syn and contractile phenotype marker gene OPN of VSMCs suggested that VSMCs phenotype were impacted, though VSMCs quantity in vitro were inhibited. Based on pathway results, we detected important member of ABC transporters. ABCC1, also known as multidrug resistance protein 1 (MRP1), mediate chemotherapy resistance via efflux cancer drugs in the presence of glutathione [8]. BEST1 is a member of Ca^2+^-activated chloride channels, holding tremendous biomedical significance [29]. Functionally, both ABCC1 and BEST1 are responsible for transporting ion, and lower expression of them may increase the accumulation of fluorine in VSMCs. The results are in a way consistent with our previous study that chloride channel 7 in fluoride treated osteoclasts was significantly lower than the control group [18]. Taken together, the above results provide more evidences regarding the response of VSMCs to fluoride.

While endothelial cells (ECs) are commonly recognized as the primary guardians against vascular diseases, it's essential to appreciate the central role that VSMCs play within the cardiovascular system. The current study provides a comprehensive metabolic profile of VSMCs subjected to fluoride exposure, unveiling a plethora of DAMs and potential research avenues. Cumulatively, these DAMs indicate deviations in amino acids metabolism, impacting the TCA cycle and acetyl-CoA synthesis, which may decrease ATP production and impair mitochondria. Furthermore, the ABC transporters pathway appears to be central to VSMCs disruption due to significant decrease in ATP synthesis, as we proposed in Fig. 5D.

Our findings undeniably pave the way for a new direction and an intriguing avenue of exploration in the realm of fluoride toxicity research and fluorosis. Nevertheless, it is essential to acknowledge that this study currently lacks robust in vivo verification of the consequences associated with inhibited protein synthesis. Precise experimental evidence is warranted to ascertain whether the toxic effects of fluoride on VSMCs stem from mitochondrial damage, TCA cycle interference, or other factors. Notably, although the concentrations of sodium on VSMCs cytotoxicity in vivo or in vitro have been reported dozens of times more than we used in this study [27, 36, 48], the researchers should consider to add sodium control group to tell the different impact between fluoride and sodium administration.

## Conclusion

This study marks a groundbreaking achievement in shedding light on the metabolites and metabolic pathways affected by fluoride in VSMCs. For the first time, we have provided a comprehensive report on these impacts and gained a deeper understanding of their implications. Notably, amino acids belonging to the "glutamate family" emerged as highly enriched components across various pathways. This underscores the significance of amino acids metabolism as a central link in fluoride-induced toxicity in VSMCs. Of particular interest is the observation that multiple pathways related to protein synthesis, and interconnected with amino acids metabolism, exhibited significant enrichment. These encompassed pathways such as ABC transporters and aminoacyl-tRNA biosynthesis, among others. While it's important to acknowledge the study's limitation in using male rats only and lacking in vivo validation of metabolites, our findings undeniably pave the way for a new direction and an intriguing avenue of exploration in the realm of fluoride toxicity research and fluorosis. In particular, whether ABC transporters is a key link in fluoride induced VSMC cytotoxicity will be the focus of in further research.

## Data Availability

Data The datasets used and/or analysed during the current study are available from the corresponding author on reasonable request.
